# Otology-Neurotology 2020 US Workforce Distribution

**DOI:** 10.1097/ONO.0000000000000007

**Published:** 2021-12-09

**Authors:** Geoffrey C. Casazza, Bradley W. Kesser, Andrew M. Strumpf, Richard K. Gurgel, George T. Hashisaki

**Affiliations:** 1 Department of Otolaryngology-Head and Neck Surgery, University of Nebraska Medical Center, Omaha, NE; 2 Department of Otolaryngology-Head and Neck Surgery, University of Virginia, Charlottesville, VA; 3 Division of Otolaryngology-Head and Neck Surgery, University of Utah, Salt Lake City, UT.

**Keywords:** Neurotology, Otolaryngology, Otology, Population distribution, Provider distribution

## Abstract

**Objective:**

Determine the geographic and per capita distribution of otology-neurotology physician-surgeons within the United States.

**Study Design:**

National database review.

**Setting:**

United States.

**Methods:**

The American Neurotology Society and ENThealth.org membership databases were queried to identify otology-neurotology physician-surgeons within the United States. Physician-surgeons were divided by regional national census areas, state, and by largest population statistical area based on 2019 US Census population estimates. The number of otology-neurotology physician-surgeons per 1 million persons was calculated for each population designation.

**Results:**

Four-hundred eighty-two physician-surgeons were identified, representing 49 states and the District of Columbia. The New England division (Connecticut, Maine, Massachusetts, New Hampshire, Rhode Island) was the most concentrated region (1.89), whereas the West-South-Central (Texas, Oklahoma, Arkansas, Louisiana) was the least concentrated region (1.23) per million persons. The highest concentration of physician-surgeons by state is within the District of Columbia (4.25), Vermont (3.21), North Dakota (2.62), Massachusetts (2.61), and New York (2.21) (per million), whereas Mississippi (0.67), Georgia (0.66), Idaho (0.56), New Mexico (0.48), and Wyoming (0.0) were the least concentrated per million persons. Increasing number of physician-surgeons was significantly correlated by state (*r*^2^ = 0.9; *P* < 0.0001), largest statistical area (*r*^2^ = 0.88; *P* < 0.0001), and census region (*r*^2^ = 0.95; *P* < 0.0005).

**Conclusions:**

Otology-neurotology physician-surgeons are evenly distributed across the United States; however, certain areas are likely underrepresented. Variability in clinical practice may account for under or overrepresentation not captured in this analysis.

## Abstract 2

Supplemental Digital Content is available in the text.

Determining the need for surgeons treating pathology of the ear and lateral cranial base is challenging. Evolving care algorithms, a changing patient population, and constant and continued advances in knowledge and technology are changing the clinical landscape in otology-neurotology. These changes are coupled with a physician-surgeon base with diverse clinical interests, surgical expertise, and varied local, regional, and national referral patterns. To understand the need and distribution of surgeons specializing in otology-neurotology, certain assumptions about the surgeon supply and subsequent demand for their services must be made. A 2013 publication attempted to estimate this by determining the number of practicing neurotologists and their distribution among hospital referral regions (1). Ultimately, this study concluded that while the distribution of specialists was closely correlated to the regional population, the estimated numbers of some procedures, specifically those of the lateral cranial base, were declining. These findings suggested that the number of neurotologists would likely exceed the projected need.

While the population growth within the United States has remained relatively constant at approximately 0.7% annual growth (2)—this growth is not evenly distributed. Areas of greater population growth are projected to be concentrated within the border states of the western, southern, and eastern United States, while other regions of the country may see their population decline, despite an overall annual increase in national population (3). Changes in life expectancy, a measure that is generally increasing, are variable among these different regions, with some seeing an overall decline in the average life expectancy (4). These demographic changes, in addition to changes in the evolution of healthcare delivery, are reshaping the expected demand for physicians—specifically for subspecialists with narrow fields of practice.

As more otolaryngology residents are entering advanced fellowship training, the discrepancy in surgeon supply and demand may continue to grow—more so in specific regions of the United States, as the number of trainees entering the work force outpaces that of established physician-surgeons leaving it (5). There are however large regions and population centers within the United States that may be underrepresented by subspecialty otology-neurotology care and identifying these regions may improve access to expert and expedient care for patients within these areas of underrepresentation. The objective of this study was to determine the local, state, and regional distributions of surgeons specializing in otology-neurotology within the United States.

## METHODS

The American Neurotology Society (ANS) and ENThealth.org membership databases were used to identify otology-neurotology subspecialty physician-surgeons within the United States. These databases were queried between April 2020 and June 2020. Only physician-surgeons practicing independently as of June 2020 were included. Trainee and senior members of the ANS, as well as foreign members practicing outside of the United States or those with a PhD as their only doctorate were excluded. The physician-surgeon’s city and state of practice were identified from the initial database search. If the location was unclear from the initial search, the National Physician-surgeon Identifier Database Registry (npidb.org) was queried to identify the physician-surgeon’s location of practice.

**FIG. 1.**
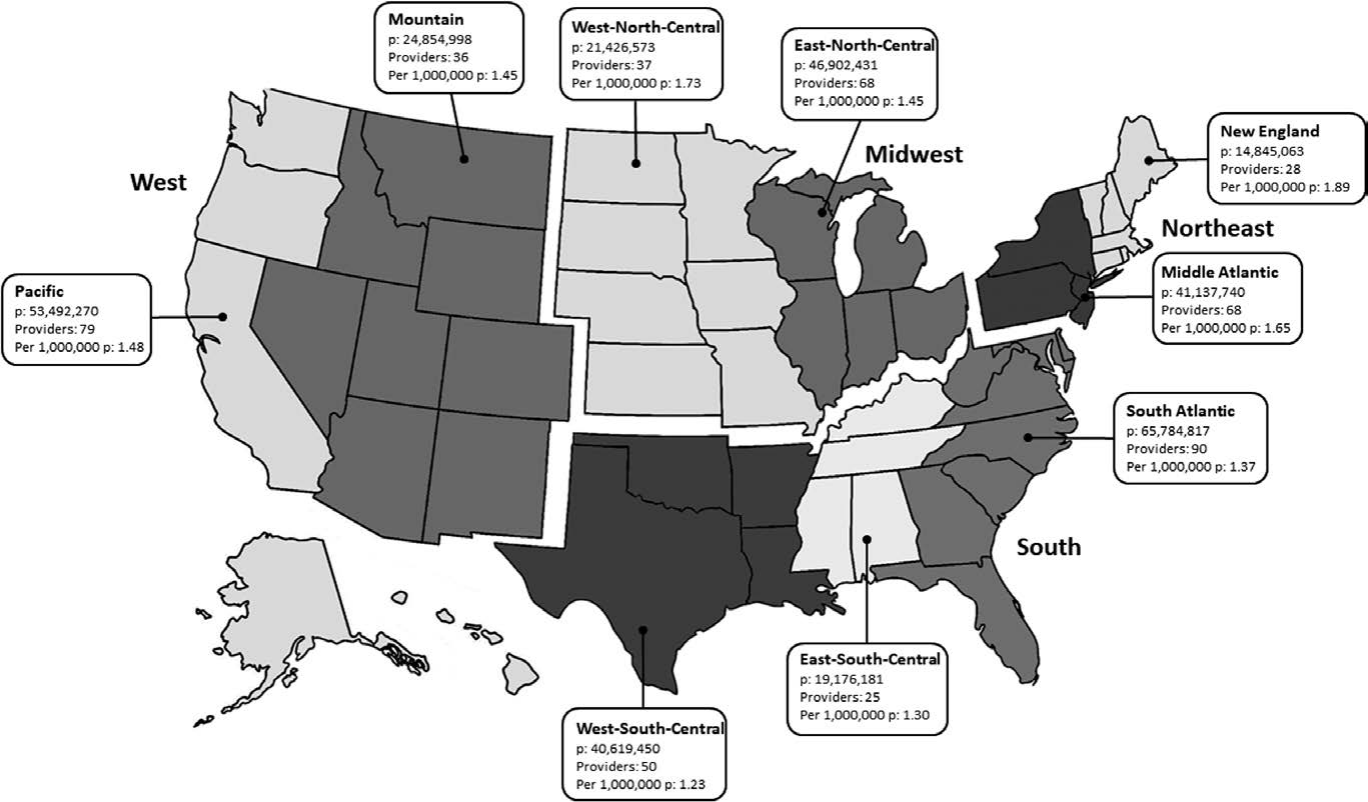
Number of physician-surgeons in each of the 4 major census regions (Northeast, South, Midwest, and West) and in each of the 9 census divisions (New England, Middle Atlantic, South Atlantic, East-North-Central, West-North-Central, East-South-Central, West-South-Central, Mountain, and Pacific). Number of physician-surgeons per million persons is listed. No. indicates number; p, persons.

Physician-surgeons were divided by US Census designations, by state, and by largest statistical area. There are 4 major statistical census regions within the United States: Northeast, South, Midwest, and West (6). The major statistical census regions are subdivided into 9 divisions (Fig. 1). Physician-surgeons were also divided into the largest statistical area—micropolitan, metropolitan, or combined—for comparison (7). The US Office of Management and Budget divides the nation’s metropolitan areas into metropolitan statistical areas (MSAs) and micropolitan statistical areas (microSAs). These are defined as having 1 or more adjacent counties or county equivalents with at least 1 urban core and an adjacent territory that has a high degree of social and economic integration (6). A MSA is defined as having at least 1 urban core with a population of at least 50,000 persons, while within a microSA, the largest urban core is at least 10,000 but less than 50,000 persons. Combined statistical areas (CSAs) are a combination of adjacent MSA and microSA that have an employment interchange of at least 15% (6). Physician-surgeons were divided by statistical area (MSA versus microSA) for comparison. If the MSA or microSA was within a CSA, the CSA was used as the physician-surgeon’s largest statistical area for comparison. All population data was based on US Census 2019 population estimates (8).

### Statistical Analysis

The acquired data were stored securely in a password-protected database and then imported to commercially available statistical software (StatPlus:macPro v. 6.1.7.0; AnalystSoft Inc., Alexandria, VA) for analysis. Basic statistical analysis was performed. Pearson correlation coefficients were calculated for number of physician-surgeons versus population. An α-level of 0.05 was used as criteria for statistical significance.

## RESULTS

Four-hundred eighty-two physician-surgeons were identified. These physician-surgeons represented 49 states and the District of Columbia.

### State Estimations

**FIG. 2.**
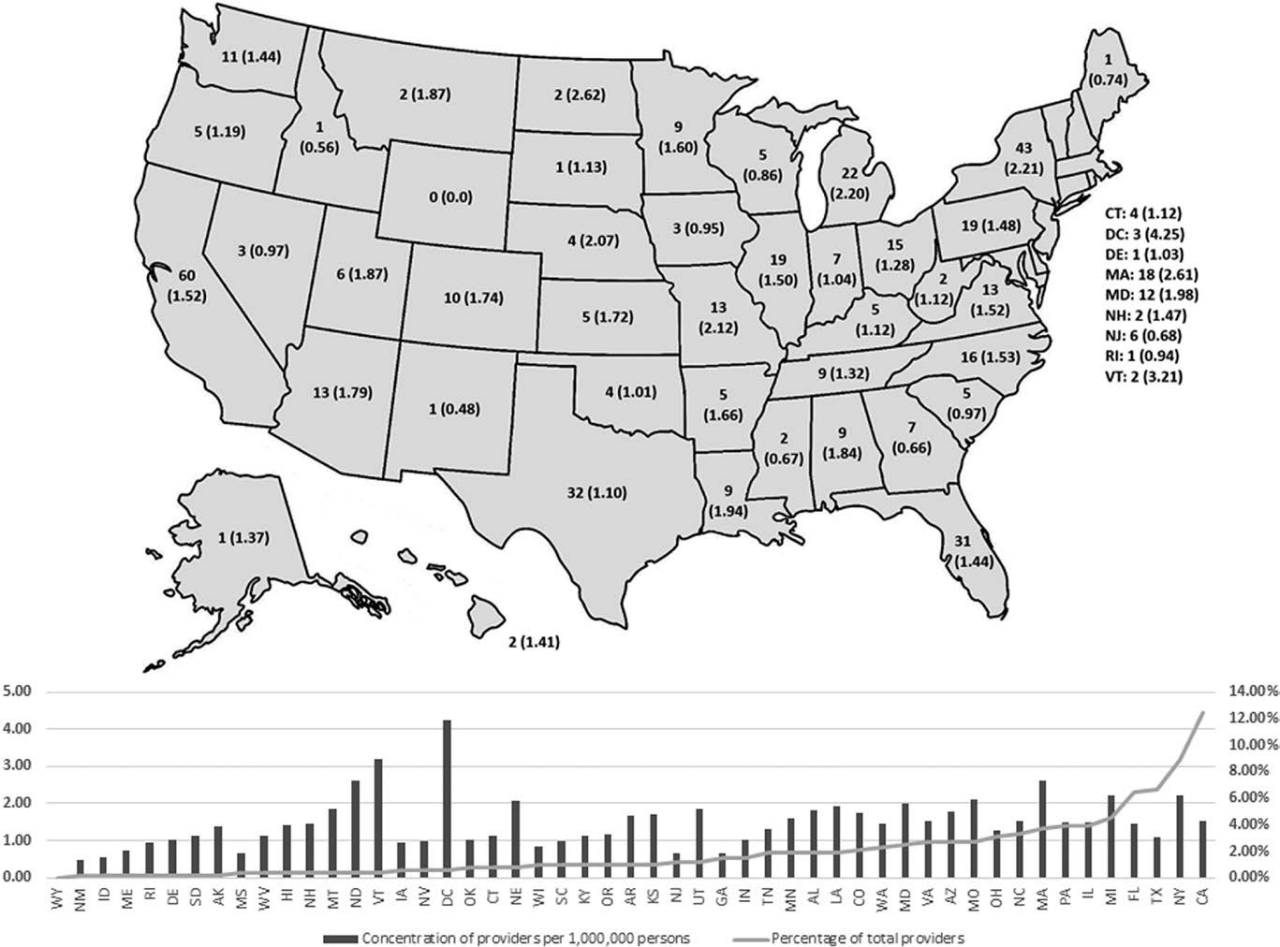
Number of physician-surgeons and number of physician-surgeons per million persons (x) by state (above). Number of physician-surgeons per state per million persons (bar) and percentage total physician-surgeons by state (line) (below).

**FIG. 3.**
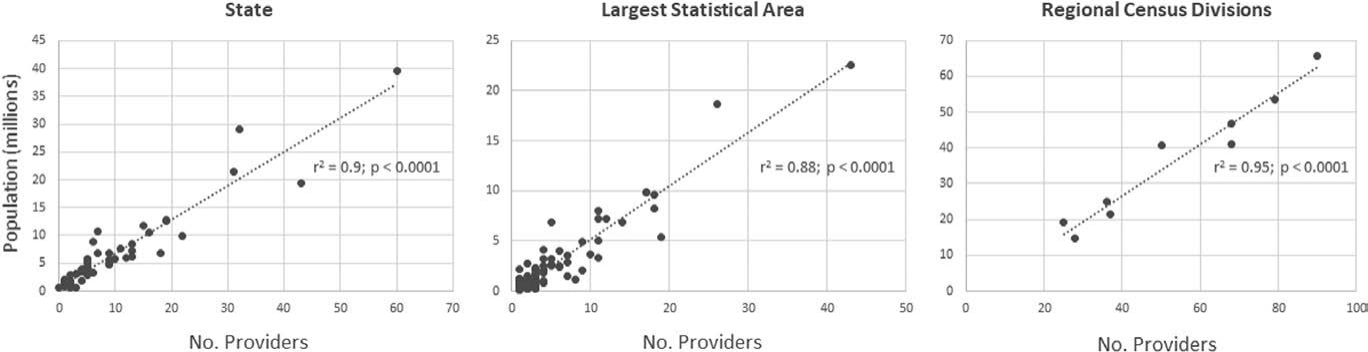
Correlation between number of physician-surgeons per million persons for state, largest statistical area, and regional census divisions. No. indicates number.

Number of physician-surgeons per state is listed in Figure 2. The highest numbers of physician-surgeons were identified in California (n = 60), New York (n = 43), Texas (n = 32), Florida (n = 31), and Michigan (n = 22). Alaska, Delaware, Idaho, Maine, New Mexico, Rhode Island, and South Dakota all had only 1 physician-surgeon identified within the state. Wyoming was the only state without a physician-surgeon. The highest concentrations of physician-surgeons per million persons, by state were within the District of Columbia (4.25), Vermont (3.21), North Dakota (2.62), Massachusetts (2.61), and New York (2.21), whereas Mississippi (0.67), Georgia (0.66), Idaho (0.56), New Mexico (0.48), and Wyoming (0.0) were the least concentrated per million persons. Increasing number of physician-surgeons was significantly correlated with increasing state population (*r*^2^ = 0.9; *P* < 0.0001) (Fig. 3).

### Largest Statistical Area Estimates

One hundred and five statistical areas had at least 1 physician-surgeon, while 458 statistical areas had no physician-surgeons. Overall, the Charlottesville, VA MSA was the most concentrated statistical area per million persons (13.72). This was followed by the Rochester-Austin, MN CSA (11.45), the Kalispell, MT microSA (9.79), the Claremont-Lebanon, NH-VT microSA (9.21), and the Missoula, MT MSA (8.36) (Table 1). The least concentrated statistical areas per million persons were the Albuquerque-Santa Fe-Las Vegas, NM CSA (0.86), the Albany-Schenectady, NY CSA (0.86), the Buffalo-Cheektowaga-Olean, NY CSA (0.83), the Atlanta–Athens-Clarke County–Sandy Springs, GA-AL CSA (0.73), the Charlotte-Concord, NC-SC CSA (0.71), and the Austin-Round Rock-Georgetown, TX MSA (0.45) (Table 2). Increasing number of physician-surgeons was significantly correlated to largest census designation population (*r*^2^ = 0.88; *P* < 0.0001) (Fig. 3). Complete physician-surgeon concentration by statistical area is reported in Supplemental Table 1 (http://links.lww.com/ONO/A1). Statistical areas with a population over 100,000 without a physician-surgeon are reported in Supplemental Table 2 (http://links.lww.com/ONO/A2).

**TABLE 1.** Ten most concentrated statistical areas (physician-surgeons per million persons)

Largest statistical area	Population	No. physician-surgeons	Per million persons
Charlottesville, VA MSA	218,615	3	13.72
Rochester-Austin, MN CSA	261,983	3	11.45
Kalispell, MT microSA	102,106	1	9.79
Claremont-Lebanon, NH-VT microSA	217,215	2	9.21
Missoula, MT MSA	119,600	1	8.36
Bloomsburg-Berwick-Sunbury, PA CSA	259,332	2	7.71
Gainesville-Lake City, FL CSA	400,814	3	7.48
Fargo-Wahpeton, ND-MN CSA	268,529	2	7.45
Birmingham-Hoover, AL MSA	1,090,435	8	7.34
Burlington-South Burlington-Barre, VT CSA	278,820	2	7.17

CSA indicates combined statistical area; microSA, micropolitan statistical area; MSA, metropolitan statistical area.

**TABLE 2.** Ten least concentrated statistical areas (physician-surgeons per million persons)

Largest statistical area	Population	No. physician-surgeons	Per million persons
Orlando-Lakeland-Deltona, FL CSA	4,160,646	4	0.96
El Paso-Las Cruces, TX-NM CSA	1,062,319	1	0.94
Tulsa-Muskogee-Bartlesville, OK CSA	1,118,150	1	0.89
Knoxville-Morristown-Sevierville, TN CSA	1,146,049	1	0.87
Albuquerque-Santa Fe-Las Vegas, NM CSA	1,158,464	1	0.86
Albany-Schenectady, NY CSA	1,167,594	1	0.86
Buffalo-Cheektowaga-Olean, NY CSA	1,204,100	1	0.83
Atlanta–Athens-Clarke County–Sandy Springs, GA-AL CSA	6,853,392	5	0.73
Charlotte-Concord, NC-SC CSA	2,797,636	2	0.71
Austin-Round Rock-Georgetown, TX MSA	2,227,083	1	0.45

CSA indicates combined statistical area; microSA, micropolitan statistical area; MSA, metropolitan statistical area.

### US Census Designation Estimations

The Northeast region was the most concentrated major statistical census region per million persons (1.71), followed by the Midwest (1.54), South (1.31), and West (1.21) regions. When the major statistical census regions were divided into their respective divisions, the New England division (Connecticut, Maine, Massachusetts, New Hampshire, Rhode Island, Vermont) was the most concentrated (1.89), whereas the West-South-Central division (Arkansas, Louisiana, Oklahoma, Texas) was the least concentrated (1.23) per million persons (Fig. 1). Increasing number of physician-surgeons was significantly correlated to regional census areas (*r*^2^ =0.95; *P* < 0.0001) (Fig. 3).

## DISCUSSION

According to the Bureau of Labor and Statistics, there are more than 36,000 surgeons within the United States (9). This means those specializing in otology-neurotology make up less than 1.5% of practicing physician-surgeons. Despite constituting a small fraction of practicing physician-surgeons, results of this current analysis demonstrate that otology-neurotology physician-surgeons are approximately evenly distributed across the country as number of physician-surgeons was strongly correlated to population within the largest statistical area, state, and region. This is despite the finding that almost 40% of physician-surgeons are practicing within 1 of 5 states (California, New York, Texas, Florida, and Michigan). Given the need for multidisciplinary collaboration and expertise, specialized resources, and a sizable patient population to support a practice, otology-neurotology physician-surgeons are more likely to be concentrated around large metropolitan areas or within major academic centers. There are, however, regions of the country, specifically within the intermountain west and southeastern United States, which may be underrepresented.

The population of the United States based on the 2019 US Census estimate was 328,239,523 persons (2). This equates to approximately 1.5 otology-neurotology physician-surgeons for every 1 million persons or a physician-surgeon for approximately every 680,000 persons. According to the 2010 US Census, the population of the United States was 308,745,538 persons. The percent increase in the US population between 2010 and 2019 was approximately 6.31%. According to the 2013 estimate by Vrabec, there were approximately 323 neurotologists practicing within the United States (1), and comparison of the results of this current analysis suggest an approximately 49.2% increase in the number of practicing otology-neurotology physician-surgeons from 2013 to this 2020 estimate.

Overall, 482 otology-neurotology physician-surgeons were identified in this current analysis. This is a tremendous increase in physician-surgeons, especially since over about the same interval, there was only an approximately 6% increase in the US population. The inclusion criteria for this current analysis were more broad then the 2013 analysis and likely included many physician-surgeons who were practicing but not included in the 2013 analysis. As this current analysis relied on multiple databases to ascertain otology-neurotology physician-surgeons and included those who may be predominately otologic surgeons, the absolute number of physician-surgeons is much higher than previously reported; however, it is likely inflated somewhat by a broader inclusion criteria. Regardless, there has been a significant increase in number of physician-surgeons over the last decade.

The Northeastern region was the most concentrated physician-surgeon region in the United States. This region is anchored by some of the most populous metropolitan areas of the country including, New York City, Boston, and Philadelphia. Many of the states and statistical areas within this region are among the most concentrated in the country based on findings in this analysis. California, although having the highest absolute number of physician-surgeons by a significant margin, was only slightly above the average in otology-neurotology physician-surgeon concentration—although most physician-surgeons were concentrated around 1 of 3 areas. The Mountain, East-South-Central, and West-South-Central regions were the least concentrated in terms of otology-neurotology physician-surgeons. The Mountain region contains the 3 least concentrated states in terms of otology-neurotology physician-surgeons (Idaho, New Mexico, and Wyoming). These states however are also among the least populated states based on 2019 census estimates. It is likely that given the population distribution within these states, a single otology-neurotology physician-surgeon may be adequate to provide care. Within the Southern region, Georgia and Mississippi were also among the least concentrated. Georgia borders and is near to many states with higher concentrations of physician-surgeons possibly explaining the low concentration of physician-surgeons identified in the state. Surprisingly, only 6 otology-otology physician-surgeons were identified in New Jersey placing it among the least concentrated states—even despite its inclusion within the concentrated Northeastern region. This may be due to the states proximity to large metropolitan areas, including New York City and Philadelphia.

Specific to the authors of this article, one of the most surprising findings was that the Charlottesville, VA MSA was the most concentrated statistical area identified in this analysis. The Charlottesville MSA has a population of only 218,000 but is anchored by a large tertiary academic hospital with 3 otology-neurotology physician-surgeons. Nine of the 10 most concentrated statistical areas have a population of less than 500,000. Seven of these 10 statistical areas are anchored by an academic tertiary care hospital with an otolaryngology residency program (Charlottesville, VA: University of Virginia; Rochester-Austin, MN: Mayo Clinic; Claremont-Lebanon, NH: Dartmouth-Hitchcock, Bloomsburg-Berwick-Sunbury, PA: Geisinger Commonwealth; Gainesville-Lake City, FL: University of Florida; Birmingham-Hoover, AL: University of Alabama, Birmingham; Burlington-South Burlington-Barr, VT: University of Vermont). The statistical areas with an otology-neurotology physician-surgeons range in population from approximately 100,000 to 22.5 million persons. While these statistical areas represent only 250 million persons across the United States many are bordering or near an area without a physician-surgeon. There is likely significant interchange among statistical areas. This may serve to distort the apparent distribution and concentration of otology-neurotology physician-surgeons within these specific areas.

The optimal patient population to support a single neurotologist has been previously reported to be approximately 1.2 million persons (1). In contrast, areas with populations of less than 500,000 persons were thought to be inadequate to support a physician-surgeon practicing the full spectrum of otology-neurotology. While these estimations can be used to identify regions that may be under or overrepresented by physician-surgeons, they are based on numerous assumptions, many of which are difficult to quantify. Patients are not bound to receive care within their immediate locale. Many of the population measures chosen in this current study to represent local, state, and regional referral patterns, are adjacent to or bordering one and other. Even those areas without a physician-surgeon (Supplemental Table 1, http://links.lww.com/ONO/A1) may be bordering or near a region or area with an otology-neurotology physician-surgeon, allowing for potential interchange and access to a provider (1). Patients may also choose to travel long distances to seek care from a specific physician-surgeon. This may be because of their expertise, patient preference, insurance limitations, or for many other reasons that may be specific to the patient or their disease process.

There have been substantial shifts in otology-neurotology over the past quarter century, and many disorders once treated with invasive surgical procedures are now managed with more conservative options. Evolution in care for cranial base tumors, including vestibular schwannomas and skull base paragangliomas, has shifted the paradigm to an increased emphasis on observation over microsurgical resection, while care for chronic vestibular disorders, such as Meniere’s disease, has deviated from inpatient to office procedures (10–14). These paradigm shifts may alter the relative distribution and concentration of physician-surgeons, as a reduction in surgical case load may lead to an excess of otology-neurotology physician-surgeons—especially as the number of new trainees is expected to continue to increase (5). In contrast, the rising incidence of other skull base disorders, including temporal lobe encephaloceles and superior semicircular canal dehiscence, and expanding indications for cochlear implants will likely continue to increase the need for otology-neurotology physician-surgeons (15). Furthermore, physician-surgeons skilled in managing basic and complex otologic disorders are needed as many otolaryngology residents do not plan to make even basic otology a part of their future practice (16). It is likely that new and future otology-neurotology physician-surgeons will face a significantly different clinical landscape than their mentors did. The increase in otology-neurotology physician-surgeons is outpacing that of the general US population (1). Ultimately, this will force innovation and advancement in new surgical treatments and technology to treat disorders of the ear and lateral cranial base.

This current analysis is dependent on many assumptions that form its foundation; it has inherent limitations due to its design, analysis, and these assumptions. Typical procedures performed by physician-surgeons included in this analysis include surgery for chronic ear disease, cochlear implants, vestibular surgery, and lateral cranial base surgery. Many of these procedures will have significant overlap with either general otolaryngology or other surgical subspecialties. The extent to which each individual physician-surgeon included in this analysis performs these procedures is unknown. Included physician-surgeons likely fall on a spectrum, from those providing only basic otologic care to those in high-volume skull base surgery practices. This is the most significant weakness of this analysis, as these variable practice patterns, clinical interests and expertise, and comfort level may affect the relative distribution and concentration of physician-surgeons included in this analysis.

While inability to incorporate each individual physician-surgeon’s scope of practice into the analysis likely remains the most significant weakness, there are other limitations of this study. Clinical practices are dynamic and physician-surgeons are not static in their positions. Results of our analysis represent the approximate distribution at the time of data collection in the year 2020. While we do expect minor changes and shifts in the distribution over time, the overall distribution will likely not significantly change; however, periodic reanalysis will be needed to look for and evaluate trends in distribution over time. Additionally, those who are not members of the ANS or the American Academy of Otolaryngology-Head and Neck Surgery were possibly not captured and included in the analysis, demonstrating another weakness of our study. For certain regions of the country where the concentration of otology-neurotology physician-surgeons is low (ie, ≤1.0 physician-surgeon per million persons), not including physician-surgeons into the analysis may significantly alter the result for that particular region. It would not, however, alter the overall findings and conclusion of our study. Another potential limitation of the study is the population measures chosen to represent various referral regions for determining the physician-surgeon density. A previous study used hospital referral regions to measure this density (1). In this updated analysis, we chose to use statistical areas to represent local referrals, states to represent statewide referrals (specifically to an academic center) and census regions to represent possible regional tertiary referrals for complex skull base pathology. Despite differences in ways to represent population measures, results of this current analysis are remarkable similar to that of the prior (1), even with an approximately 14-year increase in population. This validates the decision to use these population measures to determine physician-surgeon density. Still, results of this analysis make the assumption that patients are bound to their respective statistical area, state, or region. This is not always the case and therefore remains a significant limitation to this study. Ultimately, results of this analysis are still representative of the overall distribution of otology-neurotology physician-surgeons within the United States.

## CONCLUSIONS

Analyzing the workforce in otology-neurotology is complex. While physician-surgeons are essentially evenly distributed across the United States, the relative distribution is likely skewed towards large metropolitan areas or those areas with an academic medical center. Despite this assumption, there are areas of the United States that are underrepresented in otology-neurotology care and could be opportunities for growth. While the pace of new trainees will likely continue to outpace the retirement rate of established physician-surgeons, the increased concentration of physician-surgeons may foster development for novel interventions or procedures, which may ultimately serve to further expand the scope of otology and neurotology.

## FUNDING SOURCES

None declared.

## CONFLICT OF INTEREST

None declared.

## Data Availability Statement

Data is publicly available via www.americanneurotologysociety.com and www.census.gov.

## Supplementary Material


