# Supplementary material for: Otology-Neurotology 2020 US Workforce Distribution
Source: Otol Neurotol Open. 2021 Dec 9;1(2):e007. doi: 10.1097/ONO.0000000000000007 (PMC10969502; doi:10.1097/ONO.0000000000000007)
Supplement: Supplementary file 1 [file ono-1-e007-s001.pdf]

Largest Statistical Area	Population	No. Providers	Per million persons	Largest Statistical Area	Population	No. Providers	Per million persons
Charlottesville, VA MSA	218,615	3	13.72	Oklahoma City-Shawnee, OK CSA	1,481,542	3	2.02
Rochester-Austin, MN CSA	261,983	3	11.45	Cleveland-Akron-Canton, OH CSA	3,586,918	7	1.95
Kalispell, MT microSA	102,106	1	9.79	Nashville-Davidson–Murfreesboro, TN CSA	2,062,547	4	1.94
Claremont–Lebanon, NH-VT microSA	217,215	2	9.21	San Antonio-New Braunfels-Pearsall, TX CSA	2,571,266	5	1.94
Missoula, MT MSA	119,600	1	8.36	Pittsburgh-New Castle-Weirton, PA-OH-WV CSA	2,603,259	5	1.92
Bloomsburg-Berwick-Sunbury, PA CSA	259,332	2	7.71	New York-Newark, NY-NJ-CT-PA CSA	22,589,036	43	1.90
Gainesville-Lake City, FL CSA	400,814	3	7.48	Sacramento-Roseville, CA CSA	2,639,124	5	1.89
Fargo-Wahpeton, ND-MN CSA	268,529	2	7.45	Salt Lake City-Provo-Orem, UT CSA	2,641,048	5	1.89
Birmingham-Hoover, AL MSA	1,090,435	8	7.34	Fayetteville-Springdale-Rogers, AR MSA	534,904	1	1.87
Burlington-South Burlington-Barre, VT CSA	278,820	2	7.17	San Jose-San Francisco-Oakland, CA CSA	9,665,887	18	1.86
Logan, UT-ID MSA	142,165	1	7.03	Dayton-Springfield-Kettering, OH CSA	1,080,282	2	1.85
Morgantown-Fairmont, WV CSA	195,116	1	5.13	Seattle-Tacoma, WA CSA	4,903,675	9	1.84
Charleston-North Charleston, SC MSA	802,122	4	4.99	Tucson-Nogales, AZ CSA	1,093,777	2	1.83
Joplin-Miami, MO-OK CSA	210,691	1	4.75	Jacksonville-St. Marys-Palatka, FL-GA CSA	1,688,701	3	1.78
New Orleans-Metairie-Hammond, LA-MS CSA	1,507,017	7	4.64	Greensboro–Winston-Salem–High Point, NC CSA	1,689,151	3	1.78
Shreveport-Bossier City-Minden, LA CSA	433,046	2	4.62	Washington-Baltimore-Arlington, DC-MD-VA-WV-PA CSA	9,814,928	17	1.73
Cedar Rapids-Iowa City, IA CSA	446,137	2	4.48	Chicago-Naperville, IL-IN-WI CSA	9,825,325	17	1.73
Champaign-Urbana, IL MSA	226,033	1	4.42	Savannah-Hinesville-Statesboro, GA CSA	583,882	1	1.71
Killeen-Temple, TX MSA	460,303	2	4.34	Houston-The Woodlands, TX CSA	7,253,193	12	1.65
Raleigh-Durham-Cary, NC CSA	2,079,687	9	4.33	Augusta-Richmond County, GA MSA	608,980	1	1.64
Syracuse-Auburn, NY CSA	725,169	3	4.14	Fort Wayne-Huntington-Auburn, IN CSA	611,373	1	1.64
Omaha-Council Bluffs-Fremont, NE-IA CSA	986,007	4	4.06	Huntsville-Decatur, AL CSA	624,427	1	1.60
Lexington-Fayette–Richmond–Frankfort, KY CSA	745,033	3	4.03	Columbus-Marion-Zanesville, OH CSA	2,525,639	4	1.58
Fort Smith, AR-OK MSA	250,368	1	3.99	Harrisburg-York-Lebanon, PA CSA	1,271,801	2	1.57
Columbia-Moberly-Mexico, MO CSA	258,309	1	3.87	Portland-Lewiston-South Portland, ME CSA	646,777	1	1.55
Sioux Falls, SD MSA	268,232	1	3.73	Portland-Vancouver-Salem, OR-WA CSA	3,259,710	5	1.53
Detroit-Warren-Ann Arbor, MI CSA	5,341,994	19	3.56	Philadelphia-Reading-Camden, PA-NJ-DE-MD CSA	7,209,620	11	1.53
Greenville-Kinston-Washington, NC CSA	283,685	1	3.53	Minneapolis-St. Paul, MN-WI CSA	4,027,861	6	1.49
Wilmington, NC MSA	297,533	1	3.36	Milwaukee-Racine-Waukesha, WI CSA	2,047,966	3	1.46
Little Rock-North Little Rock, AR CSA	908,941	3	3.30	Springfield, MA MSA	697,382	1	1.43
San Diego-Chula Vista-Carlsbad, CA MSA	3,338,330	11	3.30	Los Angeles-Long Beach, CA CSA	18,711,436	26	1.39
Springfield-Jacksonville-Lincoln, IL CSA	306,399	1	3.26	Dallas-Fort Worth, TX-OK CSA	8,057,796	11	1.37
Roanoke, VA MSA	313,222	1	3.19	Hartford-East Hartford, CT CSA	1,470,083	2	1.36
Jackson-Vicksburg-Brookhaven, MS CSA	674,340	2	2.97	Louisville/Jefferson County–Elizabethtown–Bardstown, KY-IN CSA	1,489,142	2	1.34
Wichita-Winfield, KS CSA	675,126	2	2.96	Cincinnati-Wilmington-Maysville, OH-KY-IN CSA	2,280,246	3	1.32
North Port-Sarasota, FL CSA	1,063,906	3	2.82	Las Vegas-Henderson, NV CSA	2,313,238	3	1.30
Denver-Aurora, CO CSA	3,617,927	10	2.76	Charleston-Huntington-Ashland, WV-OH-KY CSA	776,694	1	1.29
Spokane-Spokane Valley-Coeur d'Alene, WA-ID CSA	734,218	2	2.72	Tampa-St. Petersburg-Clearwater, FL MSA	3,194,831	4	1.25
Rochester-Batavia-Seneca Falls, NY CSA	1,160,940	3	2.58	Boise City-Mountain Home-Ontario, ID-OR CSA	831,235	1	1.20
Anchorage, AK MSA	396,317	1	2.52	Des Moines-Ames-West Des Moines, IA CSA	877,991	1	1.14
Indianapolis-Carmel-Muncie, IN CSA	2,457,286	6	2.44	Columbia-Orangeburg-Newberry, SC CSA	963,048	1	1.04
St. Louis-St. Charles-Farmington, MO-IL CSA	2,907,648	7	2.41	Chattanooga-Cleveland-Dalton, TN-GA CSA	1,004,573	1	1.00
Kansas City-Overland Park-Kansas City, MO-KS CSA	2,501,151	6	2.40	Orlando-Lakeland-Deltona, FL CSA	4,160,646	4	0.96
Richmond, VA MSA	1,291,900	3	2.32	El Paso-Las Cruces, TX-NM CSA	1,062,319	1	0.94
Madison-Janesville-Beloit, WI CSA	892,661	2	2.24	Tulsa-Muskogee-Bartlesville, OK CSA	1,118,150	1	0.89
Phoenix-Mesa, AZ CSA	5,002,221	11	2.20	Knoxville-Morristown-Sevierville, TN CSA	1,146,049	1	0.87
Memphis-Forrest City, TN-MS-AR CSA	1,371,039	3	2.19	Albuquerque-Santa Fe-Las Vegas, NM CSA	1,158,464	1	0.86
Boston-Worcester-Providence, MA-RI-NH-CT CSA	8,287,710	18	2.17	Albany-Schenectady, NY CSA	1,167,594	1	0.86
Virginia Beach-Norfolk, VA-NC CSA	1,859,197	4	2.15	Buffalo-Cheektowaga-Olean, NY CSA	1,204,100	1	0.83
Springfield, MO MSA	470,300	1	2.13	Atlanta–Athens-Clarke County–Sandy Springs, GA-AL CSA	6,853,392	5	0.73
Grand Rapids-Kentwood-Muskegon, MI CSA	1,412,470	3	2.12	Charlotte-Concord, NC-SC CSA	2,797,636	2	0.71
Urban Honolulu, HI MSA	974,563	2	2.05	Austin-Round Rock-Georgetown, TX MSA	2,227,083	1	0.45
Miami-Port St. Lucie-Fort Lauderdale, FL CSA	6,889,936	14	2.03				

Supplemental Table 1. Complete results of largest statistical area provider concentration (providers per million persons).
CSA: combined statistical area
MSA: metropolitan statistical area
microSA: micropolitan statistical area
