# Supplementary material for: Otology-Neurotology 2020 US Workforce Distribution
Source: Otol Neurotol Open. 2021 Dec 9;1(2):e007. doi: 10.1097/ONO.0000000000000007 (PMC10969502; doi:10.1097/ONO.0000000000000007)
Supplement: Supplementary file 2 [file ono-1-e007-s002.pdf]

Largest Statistical Area	Population	Largest Statistical Area	Population	Largest Statistical Area	Population
Greenville-Spartanburg-Anderson, SC CSA	1,475,235	College Station-Bryan, TX MSA	264,728	Sioux City, IA-NE-SD MSA	144,701
Fresno-Madera-Hanford, CA CSA	1,309,368	Lynchburg, VA MSA	263,566	Flagstaff, AZ MSA	143,476
Cape Coral-Fort Myers-Naples, FL CSA	1,197,501	Harrisonburg-Staunton, VA CSA	258,084	Pottsville, PA microSA	141,359
McAllen-Edinburg, TX CSA	933,340	Hattiesburg-Laurel, MS CSA	253,330	Sumter, SC MSA	140,466
Bakersfield, CA MSA	900,202	Tuscaloosa, AL MSA	252,047	Wheeling, WV-OH MSA	138,948
Baton Rouge, LA MSA	854,884	Idaho Falls-Rexburg-Blackfoot, ID CSA	251,347	La Crosse-Onalaska, WI-MN MSA	136,616
Fayetteville-Sanford-Lumberton, NC CSA	854,826	Yakima, WA MSA	250,873	Carbondale-Marion, IL MSA	135,764
Allentown-Bethlehem-Easton, PA-NJ MSA	844,052	Monroe-Ruston, LA CSA	247,003	Eureka-Arcata-Fortuna, CA microSA	135,558
Toledo-Findlay-Tiffin, OH CSA	831,295	Redding-Red Bluff, CA CSA	245,164	Cape Girardeau-Sikeston, MO-IL CSA	135,045
South Bend-Elkhart-Mishawaka, IN-MI CSA	809,069	Lake Charles-Jennings, LA CSA	241,777	Paducah-Mayfield, KY-IL CSA	133,538
Colorado Springs, CO MSA	745,791	State College-DuBois, PA CSA	241,640	Hot Springs-Malvern, AR CSA	133,157
Mobile-Daphne-Fairhope, AL CSA	652,770	Binghamton, NY MSA	238,691	Edwards-Glenwood Springs, CO CSA	132,955
Reno-Carson City-Fernley, NV CSA	637,973	Prescott Valley-Prescott, AZ MSA	235,099	Manhattan, KS MSA	130,285
Youngstown-Warren, OH-PA CSA	637,964	Bowling Green-Glasgow, KY CSA	233,560	Bismarck, ND MSA	128,949
Lafayette-Opelousas-Morgan City, LA CSA	620,679	Topeka, KS MSA	231,969	Mankato-New Ulm, MN CSA	126,935
Myrtle Beach-Conway, SC-NC CSA	559,581	Bellingham, WA MSA	229,247	Lawton, OK MSA	126,415
Scranton–Wilkes-Barre, PA MSA	553,885	Hilton Head Island-Bluffton-Beaufort, SC MSA	222,195	Sierra Vista-Douglas, AZ MSA	125,922
Lansing-East Lansing, MI MSA	550,391	Bend-Prineville, OR CSA	222,096	Pittsfield, MA MSA	124,944
Lancaster, PA MSA	545,724	Chico, CA MSA	219,186	Farmington, NM MSA	123,958
Asheville-Marion-Brevard, NC CSA	542,821	Lima-Van Wert-Celina, OH CSA	217,454	Scottsboro-Fort Payne, AL CSA	123,139
Pensacola-Ferry Pass, FL-AL CSA	539,262	Pueblo-Cañon City, CO CSA	216,263	Goldsboro, NC MSA	123,131
Corpus Christi-Kingsville-Alice, TX CSA	535,257	Mansfield-Ashland-Bucyrus, OH CSA	216,132	Augusta-Waterville, ME microSA	122,302
Johnson City-Kingsport-Bristol, TN-VA CSA	510,851	Eau Claire-Menomonie, WI CSA	214,672	San Angelo, TX MSA	122,027
Kalamazoo-Battle Creek-Portage, MI CSA	503,706	Bloomington-Bedford, IN CSA	214,600	Altoona, PA MSA	121,829
Columbus-Auburn-Opelika, GA-AL CSA	485,590	Yuma, AZ MSA	213,787	Victoria-Port Lavaca, TX CSA	121,032
Davenport-Moline, IA-IL CSA	468,265	Houma-Thibodaux, LA MSA	208,075	Wenatchee, WA MSA	120,629
Visalia, CA MSA	466,195	Bloomington-Pontiac, IL CSA	207,165	Owensboro, KY MSA	119,440
Montgomery-Selma-Alexander City, AL CSA	461,516	Florence, SC MSA	204,911	Brunswick, GA MSA	118,779
Salisbury-Cambridge, MD-DE CSA	447,655	Johnstown-Somerset, PA CSA	203,639	Kokomo-Peru, IN CSA	118,060
Santa Maria-Santa Barbara, CA MSA	446,499	Tupelo-Corinth, MS CSA	203,079	Moses Lake-Othello, WA CSA	117,716
Brownsville-Harlingen-Raymondville, TX CSA	444,521	Hilo, HI microSA	201,513	Beckley, WV MSA	115,767
Salinas, CA MSA	434,061	Dothan-Ozark, AL CSA	198,530	Sheboygan, WI MSA	115,340
Rockford-Freeport-Rochelle, IL CSA	431,257	Jacksonville, NC MSA	197,938	Bozeman, MT microSA	114,434
Gulfport-Biloxi, MS MSA	417,665	Jackson-Brownsville, TN CSA	195,948	Cookeville, TN microSA	114,272
Macon-Bibb County–Warner Robins, GA CSA	415,405	New Bern-Morehead City, NC CSA	193,757	Quincy-Hannibal, IL-MO CSA	114,050
Appleton-Oshkosh-Neenah, WI CSA	409,881	Terre Haute, IN MSA	186,367	Anniston-Oxford, AL MSA	113,605
Peoria, IL MSA	400,561	Billings, MT MSA	181,667	Twin Falls, ID MSA	111,290
Beaumont-Port Arthur, TX MSA	392,563	El Centro, CA MSA	181,215	Roseburg, OR microSA	110,980
Tallahassee, FL MSA	387,227	Jonesboro-Paragould, AR CSA	179,185	Show Low, AZ microSA	110,924
Eugene-Springfield, OR MSA	382,067	Elmira-Corning, NY CSA	178,835	Mount Pleasant-Alma, MI CSA	110,583
Lubbock-Plainview-Levelland, TX CSA	378,684	Jackson, TN MSA	178,644	Watertown-Fort Drum, NY MSA	109,834
Saginaw-Midland-Bay City, MI CSA	376,821	St. George, UT MSA	177,556	Ogdensburg-Massena, NY microSA	107,740
Hickory-Lenoir-Morganton, NC MSA	369,711	Panama City, FL MSA	174,705	Sebring-Avon Park, FL MSA	106,221
Green Bay-Shawano, WI CSA	368,361	Abilene, TX MSA	172,060	Bluefield, WV-VA microSA	105,633
Ocala, FL MSA	365,579	Waterloo-Cedar Falls, IA MSA	168,522	Tullahoma-Manchester, TN microSA	105,216
Kennewick-Richland-Walla Walla, WA CSA	360,372	Rapid City-Spearfish, SD CSA	167,951	Decatur, IL MSA	104,009
Lincoln-Beatrice, NE CSA	357,887	Blacksburg-Christiansburg, VA MSA	167,531	Burlington-Fort Madison-Keokuk, IA-IL-MO CSA	103,775
Fort Collins, CO MSA	356,899	Kahului-Wailuku-Lahaina, HI MSA	167,417	Fond du Lac, WI MSA	103,403
Erie-Meadville, PA CSA	354,357	Altoona-Huntingdon, PA CSA	166,973	Gadsden, AL MSA	102,268
Midland-Odessa, TX CSA	348,826	Jackson, MI MSA	158,510	Grand Forks, ND-MN MSA	100,815
Evansville, IN-KY MSA	315,086	Grand Junction, CO MSA	154,210	Danville, VA microSA	100,398
Amarillo-Pampa-Borger, TX CSA	308,731	Bangor, ME MSA	152,148		
Medford-Grants Pass, OR CSA	308,431	Alexandria, LA MSA	152,037		
Clarksville, TN-KY MSA	307,820	Williamsport-Lock Haven, PA CSA	151,931		
Wausau-Stevens Point-Wisconsin Rapids, WI CSA	307,056	Wichita Falls, TX MSA	151,254		
Rocky Mount-Wilson-Roanoke Rapids, NC CSA	297,064	Jefferson City, MO MSA	151,235		
Utica-Rome, NY MSA	289,990	Traverse City, MI microSA	150,653		
Duluth, MN-WI MSA	288,732	Ithaca-Cortland, NY CSA	149,761		
Longview, TX MSA	286,657	Homosassa Springs, FL MSA	149,657		
Tyler-Jacksonville, TX CSA	285,397	Parkersburg-Marietta-Vienna, WV-OH CSA	149,250		
Crestview-Fort Walton Beach-Destin, FL MSA	284,809	Texarkana, TX-AR MSA	148,761		
San Luis Obispo-Paso Robles, CA MSA	283,111	London, KY microSA	148,123		
Laredo, TX MSA	276,652	Florence-Muscle Shoals, AL MSA	147,970		
Waco, TX MSA	273,920	Valdosta, GA MSA	147,292		
Lafayette-West Lafayette-Frankfort, IN CSA	265,401	Albany, GA MSA	146,726		

Supplemental Table 2. Largest statistical area over 100,000 population without a provider.
CSA: combined statistical area
MSA: metropolitan statistical area
microSA: micropolitan statistical area
